# Chronic exposure to indoxacarb and pulmonary expression of toll-like receptor-9 in mice

**DOI:** 10.14202/vetworld.2016.1282-1286

**Published:** 2016-11-21

**Authors:** Sandeep Kaur, C. S. Mukhopadhyay, R. S. Sethi

**Affiliations:** School of Animal Biotechnology, Guru Angad Dev Veterinary and Animal Sciences University, Ludhiana - 141 004, Punjab, India

**Keywords:** indoxacarb, lipopolysaccharide, lungs, mice, toll-like receptor-9

## Abstract

**Aim:**

Chronic exposure to indoxacarb and pulmonary expression of toll-like receptor 9 (TLR-9) in mice.

**Materials and Methods:**

In this study, healthy male Swiss albino mice (n=30) aging 8-10 weeks were used to evaluate TLR-9 expression in lungs of mice following indoxacarb exposure with and without lipopolysaccharide (LPS). Indoxacarb was administered orally dissolved in groundnut oil at 4 and 2 mg/kg/day for 90 days. On day 91, five animals from each group were challenged with LPS/normal saline solution at 80 µg/animal. The lung tissues were processed for real time and immunohistochemical studies.

**Results:**

LPS resulted increase in fold change m-RNA expression level of TLR-9 as compare to control, while indoxacarb (4 mg/kg) alone and in combination with LPS resulted 16.21-fold change and 29.4-fold change increase in expression of TLR-9 m-RNA, respectively, as compared to control. Similarly, indoxacarb (2 mg/kg) alone or in combination with LPS also altered TLR-9 expression. Further at protein level control group showed minimal expression of TLR-9 in lungs as compare to other groups, however, LPS group showed intense positive staining in bronchial epithelium as well as in alveolar septal cells. Indoxacarb at both doses individually showed strong immuno-positive reaction as compare to control, however when combined with LPS resulted intense staining in airway epithelium as compare to control.

**Conclusion:**

Chronic oral administration of indoxacarb for 90 days (4 and 2 mg/kg) alters expression of TLR-9 at m-RNA and protein level and co-exposure with LPS exhibited synergistic effect.

## Introduction

Pesticides, hybrid category of chemicals, are the most effective means to significantly enhance agricultural productivity and crop yields by protecting plants from pests [1]. However, their entry into food chain is affecting human and livestock health by inducing immunomodulations [2]. Three million cases of pesticide poisoning, nearly 220,000 fatal, occur worldwide every year [3]. Indoxacarb is a new oxadiazine pesticide and endeavor its pesticidal action via voltage-dependent sodium channels [4]. Indoxacarb exposure results acute lung injury and high permeability pulmonary edema which is imputed to generation of an oxidant during indoxacarb metabolism [5].

Toll-like receptors (TLRs) are one of the most members of the innate immune system that play an important role to protect against invading pathogens and modulate the induction of inflammation [6]. TLR-9 is a membrane-bound receptor that is primarily accompanied with endosomes [7] and recognizes non-methylated CpG sequences of bacterial DNA [8]. It is expressed predominantly in immune cells such as peripheral blood leukocytes and in various lung cells [9]. TLR-9 has been proved to regulate [10,11], prevent [12] or modify [13] lung inflammatory responses and promote leukocyte migration and transcription of inflammatory cytokine genes [14].

Endotoxins are frequently available in the environment especially agricultural settings [15,16] and endotoxin and pesticide interaction increases toxicity of various pesticides [17]. To the best of our knowledge, no data on TLR-9 expression with indoxacarb and its combination with endotoxin have been reported. Since there remains possibility that animals and humans may get co-exposures of pesticides and endotoxins, so we tested hypothesis in a mouse model that indoxacarb exposure along with endotoxin may alter the expression of TLR-9.

## Materials and Methods

The experiment was conducted after approval by Institutional Animal Ethics Committee, Guru Angad Dev Veterinary and Animal Sciences University (GADVASU), Ludhiana. Swiss Albino mice (n=30) aging 8-10 weeks were obtained from Lala Lajpat Rai University of Veterinary and Animal Science, Hisar, Haryana. The animals were maintained in small animal colony of GADVASU under controlled conditions (22±2°C, 50% humidity, 12-h photoperiod) in the animal house for 7 days to allow acclimatization prior to experiments. Mice were provided feed (Ashirwad Industries, Chandigarh, India) and drinking water *ad libitum*.

### Chemicals

Indoxacarb (CAS no144171-61-9) PESTANAL with purity level of 99.9%, lipopolysaccharide (LPS) from *Escherichia coli* (CAS no L3129) were obtained from Sigma-Aldrich, Bengaluru, India. The others chemicals included Trizol reagent (Life Technologies), c-DNA first strand synthesis kit (Thermo Scientific, USA), TLR-9 primary antibody (IMGENEX-3051), and secondary antibody (DAKO).

### Doses

Indoxacarb doses (2 and 4 mg/kg/day) used in this study are above no observed adverse effect level, i.e., 1 mg/kg/BW.

### Experimental design

Mice were randomly divided into two treatments (IC4 and IC2) and one control group (n=10; each group). Treatment groups were administered indoxacarb orally at 4 mg/kg/day (IC4 group) and 2 mg/kg/day (IC2 group) dissolved in groundnut oil for 90 days. Control group was administered groundnut oil for 90 days. At the end of experiment five animals from all the groups were challenged with LPS (80 µl/animal) by intranasal route. The remaining animals were challenged with normal saline solution (NSS) at 80 µl/animal by the same route. After 9 h of LPS/NSS challenge, all the animals were anesthetized by intraperitoneal administration of xylazine and ketamine cocktail (0.1 µl/10 g of body weight) and humanely sacrificed.

### Tissue collection

The left lung was collected and fixed in 4% paraformaldehyde solution for 12-16 h at 4°C for histopathology and immunohistochemistry. Right lung from each animal was stored in RNA later solution for detection of expression of TLR-9 m-RNA. Further left lung tissue samples stored in paraformaldehyde were processed for paraffin block preparation to obtain 5 µm thick paraffin sections. The sections were obtained on clean poly-L-Lysine coated slides and were subjected to immunohistochemical staining to localize immmuno-positive TLR-9 expression.

### Real-time quantitative PCR analysis

Lung tissues (100 mg), kept in RNA later solution, were used for RNA extraction. Total lung RNA was extracted using Trizol Reagent (Life Technologies) according to the manufacturer’s instructions. Total RNA was determined by OD260/280 measurements with spectrophotometer (Thermo Fischer Scientific). The concentration of total RNA varied in different samples. The amount of total RNA used for c-DNA synthesis was adjusted to 400 ng/µl for each sample. Further, cDNA was synthesized from total RNA using first-strand cDNA synthesis kit (Thermo Fischer Scientific, USA) as per manufacturer’s Instruction and qPCR was performed on a Prism 7500 fast PCR system (Bio-Rad Inc., USA). mRNA expression was assayed using the primer forward 5’-TCACAGGGTAGGAAGGCA-3’ and reverse 3’-GAATCCTCCATCTCCCAACA-5’- for TLR-9 [18] and forward 5’- GCA CCA CAC CTT CTA CAA TG -3’ and reverse 3’-TGC TTG CTG ATC CAC ATC TG -5’ for β-actin [19]. Amplification of TLR-9 mRNA required an initial denaturation step at 95°C for 5 min. Temperature cycling consisted of 35 cycles of denaturation at 95°C for 10 s, annealing at 55°C for 35 s, and elongation at 72°C for 40 s. Transcript levels were normalized by comparison with β-actin.

### Immunohistochemistry

The immunohistochemistry was performed as described earlier [20]. Briefly tissue sections were de-paraffinized and rehydrated. The tissue peroxidases were inactivated with 0.5% H_2_O_2_ in phosphate buffered saline (PBS) for 20 min. Tris-ethylene-diamine-tetraacetic acid buffer was used to unmask antigen-binding sites (30 min) followed by incubation with 1% bovine serum albumin in PBS (30 min) to prevent non-specific binding. Then, the tissues were incubated with the primary antibody for TLR-9 (IMG-3051, IMGENEX; dilution 1:800) followed by appropriate secondary antibody (DAKO catalogue no# R0270 dilution 1:100). VECTOR VIP Peroxidase Substrate Kit (Vector Laboratories, Burlingame, CA) was used for color development followed by counterstaining with hemtoxylin (Vector Laboratories).

### Statistical analysis

The fold change in expression of the TLR-9 gene was determined using the ΔΔCt method [21]. The expression in the control group was used as calibrator for rest of the samples.

## Results

### TLR-9 mRNA expression

**Figure-1:**
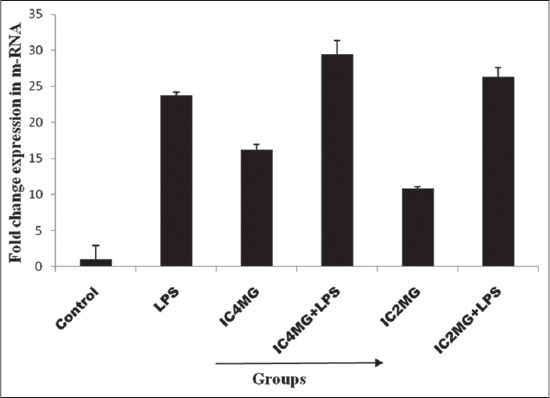
Fold change expression of toll-like receptor-9 mRNA following oral exposure to indoxacarb (4 and 2 mg/kg) for 90 days with and without lipopolysaccharide. Values are expressed as mean±standard error.

LPS challenge and indoxacarb (4 mg/kg) resulted 23.7- and 16.21-fold increase in the TLR-9 mRNA expression compared to control, respectively (Figure-1 and Table-1). Further, indoxacarb (4 mg/kg) in combination with LPS resulted 29.4-fold increases in m-RNA expression of TLR-9 compared to control and LPS (Figure-1 and Table-1). Similarly, indoxacarb (2 mg/kg) resulted 10.8 increases in fold change as compare to control while in combination with LPS showed 26.7 increases in fold change as compared to control and LPS.

**Table 1:** Fold change expression of TLR-9 mRNA following oral exposure to indoxacarb (4 and 2 mg/kg) for 90 days with and without LPS.

Group	TLR-9 average CT	β-actin average CT	∆CT (CT of TLR-9 - CT of beta actin)	∆∆CT (∆CT of exposed-∆CT of control)	Fold difference in TLR-9 (exposed) relative to control
Control	29.97±1.80	27.34±1.14	−0.52	0.00	1
LPS	26.10±1.14	29.12±0.54	−3.02	−4.88	23.7
IC4 mg	27.04±1.07	29.22±2.15	−2.18	−4.07	16.21
IC4 mg+LPS	27.33±1.67	30.55±1.53	−3.22	−4.92	29.4
IC2 mg	28.65±0.77	30.10±2.14	−1.57	−3.43	10.8
IC2 mg+LPS	27.47±0.83	30.57±0.92	−3.10	−4.78	26.3

Values are expressed as mean±SE. SE=Standard error, LPS=Lipopolysaccharide

### Immunohistochemistry

**Figure-2:**
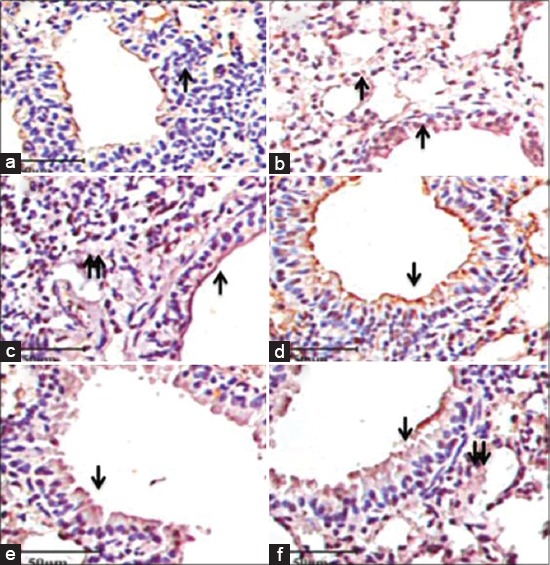
Lung tissue showing expression of toll-like receptor-9 immunopositive reactivity in airways epithelium (single arrow) and alveolar septal cells (double arrow) in control (a), lipopolysaccharide (LPS) (b), indoxacarb (4 mg/kg) (c), indoxacarb (4 mg/kg) and LPS (d) indoxacarb (2 mg/kg) (e) and indoxacarb (2 mg/kg) and LPS (f) groups (IHC×40).

In this study, control group showed minimal expression of TLR-9 in lungs as compare to other groups, however LPS group showed intense positive staining in bronchial epithelium as well as in alveolar septal cells (Figure-2a and b). Indoxacarb (4 mg/kg) exhibited strong reaction compared to control, however when combined with LPS resulted intense staining in airway epithelium as compare to indoxacarb (4 mg/kg) alone as well as control and other groups (Figure-2c and d). Indoxacarb (2 mg/kg) showed positive staining in the airway epithelium of lungs exposed for 90 days orally, however staining was intensified in airway epithelium when combined with LPS (Figure-2e and f).

## Discussion

In the present investigation, effect of oral administration of indoxacarb alone or in combination with LPS was studied in a mouse model. We report first data on pulmonary expression TLR-9 in mice following exposure to indoxacarb with and without LPS. The data from this first study on pulmonary effects of indoxacarb showed altered expression of TLR-9 at mRNA and protein level following exposure to indoxacarb alone or in combination with LPS.

Lung inflammation is regulated through activation of innate immune system comprised TLRs such as TLR-4 and TLR-9 that bind to LPS and CpG molecules, respectively [22-24]. TLR-9, under disease conditions, recognizes endogenous DNA such as mitochondrial DNA (mtDNA) [25-27]. Most of the data on TLR-9 expression has come from isolated and cultured cells and has generally been focused on the mRNA expression of TLR-9 [28-31]. In the present setting of indoxacarb poisoning, there was an increase in TLR-9 mRNA expression at both individual doses of indoxacab. We speculate that mtDNA could be a strong activator of TLR-9 and may trigger a subsequent signaling cascade. Further, LPS is known to increase TLR-9 mRNA expression in the lung [32] as observed in the present investigations. Macrophages express TLRs such as TLR-4 and TLR-9 and use these molecules to sense microbial molecules [33,34]. There was increased expression of TLR-9 mRNA and protein in these cells [35]. In this study, indoxacarb at both doses individually and when combine with LPS resulted increase in TLR-9 m-RNA expression suggesting a synergistic effect of the indoxacarb and LPS on TLR-9 mRNA expression.

In the present investigations, oral treatment with indoxacarb for 90 days showed increased airway epithelial and vascular endothelial expression of TLR-9. Indoxacarb treatment also increased the number of septal cells expressing TLR-9. There was minimal expression of TLR-9 in lungs in control group as compare to other groups, however LPS and indoxacarb (4 and 2 mg/kg) exhibited intense positive staining in brochial epithelium as well as in alveolar septal cells. TLR-9-positive septal cells were increased during the chronic obstructive pulmonary disease as compare to normal human lungs [9]. Further indoxacarb when combined with LPS showed intense staining in airway epithelium as well as in septal cells. Similarly, fipronil exposure resulted TLR-9 staining in septa, airway epithelium and blood vessels in the lungs of mice [36]. The data taken together suggest increased TLR-9 immunopositive reaction following exposure to indoxacarb alone or in combination with LPS.

We may speculate that indoxacarb exposure at both doses recruit cells in the airways and alveolar septa. Many of these recruited inflammatory cells expressed TLR-9 mRNA and protein. Although we did not identify these specific cells, work by others has suggested that these cells could include myeloid dendritic cells [37], eosinophils [38] and neutrophils [29], all of which express TLR-9 and may account for the positive immunopositive staining of lung cells.

## Conclusion

We conclude that oral administration of indoxacarb for 90 days (4 and 2 mg/kg) alters TLR-9 expression at m-RNA and protein level. We found a possible synergistic effect of indoxacarb and LPS on the pulmonary expression of TLR-9, which definitely requires further investigations.

## Authors’ Contributions

RSS designed the experiment, organized sample collection and CSM helps in statistical analysis. Experiment was performed by SK under the supervision of RSS and CSM.
